# Targeting steroid receptor co-activators to inhibit breast cancer stem cells

**DOI:** 10.18632/oncoscience.469

**Published:** 2018-08-28

**Authors:** Thu H. Truong, Carol A. Lange, Julie H. Ostrander

**Affiliations:** Departments of Medicine (Division of Hematology, Oncology, and Transplantation) and Pharmacology, Masonic Cancer Center, University of Minnesota, Minneapolis MN 55455 USA

**Keywords:** PELP1, AIB1, SRC-3, cancer stem cells, breast cancer

Treatment of invasive breast cancer involves surgical removal of the tumor with the addition of radiation, chemotherapy, and endocrine therapy depending on the stage and clinical subtype (i.e., ER+, HER2+, triple negative). While these current treatments are effective for most women and result in a 5-year survival rate over 89%, approximately 30% of patients experience disease recurrence. As recurrence is often associated with resistance to chemotherapy and/or endocrine therapy and metastasis to distant organs (e.g., bone, brain, and lung), this remains a significant health burden for women. Notably, up to 40% of women with ER+ tumors exhibit *de novo* or acquired resistance to endocrine therapies and eventually progress to metastatic disease. Hence, there is a critical need to identify novel therapeutic strategies to target breast cancer progression and prevent the emergence of therapy-resistant disease.

Cancer stem or stem-like progenitor cells (CSC) are non-proliferative and frequently exist as a minority sub-population of cells in therapy-resistant tumors [1]. CSCs form colonies in serum-free suspension culture (i.e., tumorspheres), express specific stem cell markers (e.g., ALDH+ and CD44+/CD24- populations in breast tumors), form tumors in *in vivo* limiting dilutions assays, exhibit enhanced resistance to chemo- and endocrine therapeutic agents, and express markers of epithelial to mesenchymal transition (EMT). In breast cancer, cells exhibiting ALDH1 expression have been shown to correlate with poor prognosis. Moreover, breast CSCs have been shown to contribute to increased risk of breast cancer development, disease progression, and formation of metastases [1]. Their ability to survive and expand after treatment allows CSCs to evade standard chemo- and endocrine therapies aimed at rapidly dividing cancer cells and to sustain tumor re-growth. These factors make breast CSCs an attractive therapeutic target given their ability to self-renew and expand after conventional therapies have eliminated most non-CSCs.

Growing evidence has demonstrated the role of hormones and steroid hormone receptors (SR) in promoting CSC expansion in breast tumors [1]. However, recent studies have also implicated SR co-activators, such as AIB1 (amplified in breast cancer 1; also known as SRC-3 or NCOA3), as mediators of stem/progenitor cell formation. AIB1 was shown to maintain embryonic stem cell self-renewal and reprogramming by upregulating gene programs involved in stem cell biology and pluripotency (i.e., *Oct4-Sox2-Nanog* pathway) [2]. A separate study from Rohira *et al*., demonstrated that AIB1 drives formation of cancer stem-like cells and supports tumor outgrowth in breast cancer models [3]. Interestingly, this study demonstrated enhanced expression of *Oct4*, *Sox2*, and *Nanog*. This work also described the utility of SI-2, a small molecule designed to inhibit AIB1. SI-2 treatment decreased AIB1-induced CSC formation in breast cancer cell and xenograft models. Taken together, these studies indicate that gene programs mediating stem/progenitor cell pathways can be regulated in both normal and cancer cell models by AIB1. This highlights an underlying theme in cancer biology, whereby cancer cells are able to hijack normal cell processes to promote tumor growth.

We recently reported that cytoplasmic PELP1/AIB1 complexes function to promote advanced breast cancer phenotypes, specifically the outgrowth of CSCs [4]. PELP1 (proline, glutamic acid, and leucine rich protein 1) is overexpressed in approximately 80% of invasive breast tumors. PELP1 dynamically shuttles between the nucleus and cytoplasm, but is primarily nuclear in normal breast tissue where it serves as a SR co-activator. However, altered localization of PELP1 to the cytoplasm is an oncogenic event that promotes breast cancer initiation and progression [5]. Our study sought to identify interacting partners unique to cytoplasmic PELP1. We identified AIB1 as a novel binding partner of cytoplasmic PELP1 in both normal and breast cancer cell models. Cytoplasmic PELP1 expression elevated basal phosphorylation levels (i.e., activation) of AIB1, enhanced ALDH+ tumorsphere formation, and upregulated target genes involved in cell survival (e.g., *AMIGO2*, *NELL2*). Direct manipulation of AIB1 levels using shRNA abrogated cytoplasmic PELP1-induced tumorsphere formation and down-regulated cytoplasmic PELP1-specific target genes. In addition, we were able to utilize SI-2 (AIB1 inhibitor) to block the PELP1/AIB1 protein-protein interaction and decrease cytoplasmic PELP1-induced tumorsphere formation. Furthermore, *in vivo* syngeneic tumor studies showed that PELP1 knockdown in a murine-derived MMTV-AIB1 tumor cell line increased the duration of survival for tumor-bearing mice. Our results suggest that inhibiting the cytoplasmic PELP1/AIB1 signaling complex may provide a means to specifically target the breast CSC population.

Indeed, PELP1 itself is emerging as a viable therapeutic target and biomarker for women with breast cancer. Pre-clinical studies have illustrated that peptidomimetics and small molecule inhibitors can specifically disrupt PELP1 protein-protein interactions [6]. Our study identified the cytoplasmic PELP1/AIB1 complex as a novel protein-protein interaction that could be selectively targeted by potent inhibitors (i.e., the AIB1 inhibitor SI-2). Thus, it is possible to envision that the PELP1/AIB1 complex could be targeted using a peptidomimetic approach or small molecule inhibitors that specifically target either PELP1 or AIB1. Furthermore, studies have shown that PELP1 and AIB1 independently mediate endocrine therapy resistance. Acquired resistance to endocrine therapies remains a major clinical problem. Therefore, inhibitors capable of selectively targeting cytoplasmic PELP1 or its interacting partners (e.g., AIB1) that promote therapy resistance would provide a means to target breast CSCs and could be used in lieu of, or in conjunction with, endocrine therapies.

Breast CSCs represent a small minority of the total cell population, which makes them inherently more difficult to specifically target by standard chemo- or endocrine therapies. It is crucial to identify the intracellular pathways and gene programs involved in driving breast CSC survival and expansion. Human breast CSC populations respond to both autocrine and paracrine signals and are SR+ [7]. Notably, our prior studies demonstrated that PELP1, ER, and PR-B form constitutive signaling and transcriptional complexes [8]. Namely, PR-B was required for estrogen-stimulated regulation of novel ER/PR/PELP1-target gene sets significantly associated with breast cancer progression to endocrine resistance. PR or PELP1 inhibition restored tamoxifen sensitivity. Both AIB1 and PELP1 are potent SR co-activators. We speculate that PELP1/AIB1 complexes represent an essential “node” for regulation of stem cell biology and associated gene programs by SRs that are hyper-activated in the context of cytoplasmic PELP1/AIB1 complexes. PELP1-driven signaling to nuclear transcription factors, including SRs, represents a likely mechanism by which these molecular partners function together to drive SR+ breast cancer progression, breast CSC biology, and therapy resistance. Targeting reversible events that drive advanced breast cancer biology (i.e. other than proliferation) is an unmet need that has the potential to extend patient lives and improve well-being.
